# Cancer Chemopreventive Potential of *Claoxylon longifolium* Grown in Southern Thailand: A Bioassay-Guided Isolation of Vicenin 1 as the Active Compound and *In Silico* Studies on Related *C*-Glycosyl Flavones

**DOI:** 10.3390/molecules30153173

**Published:** 2025-07-29

**Authors:** Chuanchom Khuniad, Lutfun Nahar, Anupam D. Talukdar, Rajat Nath, Kenneth J. Ritchie, Satyajit D. Sarker

**Affiliations:** 1 Centre for Natural Products Discovery (CNPD), School of Pharmacy and Biomolecular Sciences, Liverpool John Moores University, Liverpool L3 3AF, UK; k.j.ritchie@ljmu.ac.uk; 2 Department of Thai Traditional Medicine, Faculty of Health and Sports Science, Thaksin University, Phathalung 93210, Thailand; 3 Laboratory of Growth Regulators, Palacký University and Institute of Experimental Botany, The Czech Academy of Sciences, Šlechtitelů 27, 78371 Olomouc, Czech Republic; 4 Department of Life Science and Bioinformatics, Assam University, Silchar 788011, India; anupam@bioinfoaus.ac.in; 5 Department of Biotechnology and Microbiology, School of Natural Sciences, Techno India University, Agartala 799004, India; rajatnath17@gmail.com

**Keywords:** *Claoxylon longifolium*, Euphorbiaceae, AREc32 cell line, bioassay-guided solation, cancer chemoprevention, flavone *C*-glycoside, Nrf2 activation, vicenins

## Abstract

*Claoxylon longifolium* (Euphorbiaceae) is an indigenous vegetable that has been used in southern Thai traditional medicine and cuisine. A bioassay-guided approach was adopted to investigate the phytochemicals and chemopreventive potential of *C. longifolium* leaves and stems. Phytochemical investigation of the active MeOH fractions afforded six known compounds, including caffeic acid (**1**), isovitexin (**2**), and vicenins 1–3 (**3–5**) from leaves and hexadecanoic acid methyl ester (**6**) from stems. Their structures were determined by spectroscopic means. Ten constituents were tentatively identified from the oily fractions of stems by GC-MS. Non-cytotoxic concentrations of compounds **1**–**6** were identified using the MTT cell viability assay. The ability of compounds **1**–**6** at non-cytotoxic concentrations to induce Nrf2 activation, correlating to their potential chemopreventive properties, was determined using a luciferase reporter assay in the AREc32 cell line. Only vicenin 1 (**3**) was considered to be a potent chemopreventive compound, as it increased luciferase activity by 2.3-fold. In silico studies on compounds **2**–**5** and vitexin (**16**) revealed the potential of these compounds as cancer chemopreventive and chemotherapeutic agents. This study provides the first report on the chemopreventive properties of *C. longifolium*. All identified and isolated compounds are reported here for the first time from this species.

## 1. Introduction

In consideration of global cancer incidence and mortality rates reported by the International Agency for Research on Cancer (IARC), cancer is a major threat to human health worldwide [1]. As of 2022, there have been almost 20 million new cancer cases. Furthermore, 16.8% of all deaths (9.7 million) were cancer deaths, which makes it the second leading cause of death worldwide behind cardiovascular diseases. Almost one-half of all new cases (49.2%) and the majority (56.1%) of cancer deaths globally were estimated to occur in Asia in 2022. Hence, Asia has the highest burden of cancer compared to the other regions in the world. According to the GLOBOCAN 2022 database, created by the IARC, the number of new cancer cases is projected to reach 35 million by 2050, representing a 77% increase from 2022 [1]. Therefore, the search for new alternative cancer prevention and treatment options is still necessary.

Cancer chemoprevention is defined as the use of “nontoxic” natural or synthetic chemical agents to inhibit, slow down, or reverse the process of carcinogenesis by blocking or suppressing specific molecular events and signalling pathways associated with cancer development [2]. The goal of cancer chemoprevention is to decrease cancer incidence and concurrently reduce treatment-related adverse effects and mortality rates, making it an effective strategy for cancer control and management [3,4].

Nuclear factor erythroid 2-related factor 2 (Nrf2) is a cytoprotective transcription factor that regulates the expression of many cellular antioxidants and detoxification enzymes [5]. Nrf2 binds to the antioxidant response element (ARE) in the promoter regions of many cytoprotective genes, thereby promoting their expression. Thus, the Nrf2-ARE signalling pathway is recognized as a potential molecular target for cancer chemoprevention because the activation of this pathway induces the production of a battery of cytoprotective and detoxifying genes that may be a promising approach to prevent or suppress carcinogenic progression [6,7]. To date, numerous natural products that exert chemopreventive properties have been discovered as Nrf2 activators such as curcumin, diallyl sulfide, resveratrol, sulforaphane and *tert*-butylhydroquinone (*t*BHQ) [8,9,10].

**Figure 1:**
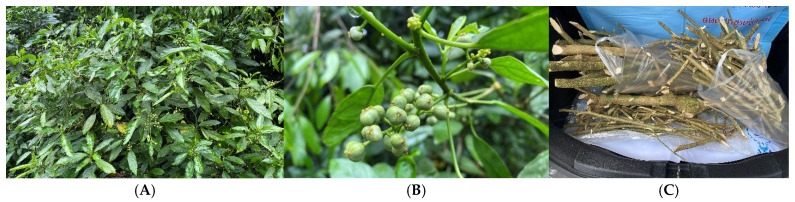
Young plant (**A**), fruits (**B**) and stems (**C**) of *C. longifolium* (photograph taken by the author C. Khuniad in 2021).

**Figure 2:**
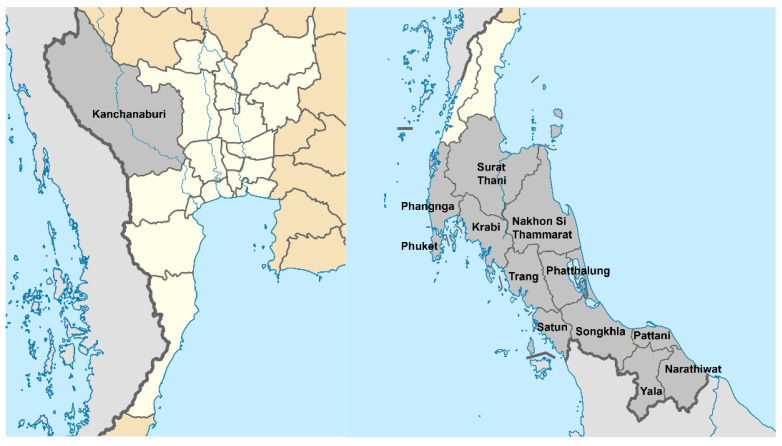
Contribution map of *C. longifolium* in central (**left**) and southern (**right**) Thailand (light colored areas indicate major sites for this plant).

Thai traditional medicine (TTM) uses many natural products to treat diseases. Even though modern medicine has made noteworthy progress, many people who live in rural communities of southern Thailand still rely on traditional healers and medicinal plants for their primary healthcare needs [11,12]. Importantly, Thailand is a biodiversity-rich country with more than 11,000 different plant species documented in the Flora of Thailand [13], many of which have medicinal properties and could be a potential source for cancer chemopreventive agents. *Claoxylon longifolium* (Blume) Endl. ex Hassk. (Euphorbiaceae), locally known as “Tamad” or “Phak wan chang” (Figure 1) is native to Northeast India, from Southeast Asia to Yunnan of southwestern China, throughout West Malaysia up to Borneo, Java and New Guinea [14,15]. The distribution of this species in Thailand is in the southern peninsula, except Kanchanaburi province (Figure 2) [15,16].

In Thailand, Malaysia, and the Philippines, the young shoots and leaves of this plant are used as vegetables and food ingredients [14,17,18]. Folk healers in southern Thailand use the whole plant to treat menstrual fever and leaves to treat blurred vision [19]. Moreover, decoction of leaves/roots can be drunk as tonic and restorative remedies [20,21]. Interestingly, this species is used by traditional Malay midwives as an ingredient in an herbal bath for postpartum recovery and rejuvenation therapy [22]. Preliminary phytochemical screening of *C. longifolium* leaves revealed the presence of alkaloids, flavonoids and terpenes [23,24]. Hu et al. [25] isolated nine phenolic compounds, including cleomiscosin A, cleomiscosin C, ficusal, scopoletin, ferulaldehyde, isovanillic acid, protocatechuic acid, vanillin and *p*-hydroxybenzaldehyde from the stems of *C. longifolium*. To the best of our knowledge, there is no published work on pharmacological studies available in any well-known scientific database. Therefore, based on the traditional uses and previously reported phytochemical studies of *C. longifolium*, it was hypothesised that this plant could have chemopreventive activity, and accordingly, this study aimed to investigate the phytochemicals and chemopreventive effect of *C. longifolium* leaves and stems growing in southern Thailand. This paper reports, for the first time, the bioassay-guided isolation of vicenin 1 (**3**) as a potential chemopreventive agent from the methanolic extract of the leaves of *C. longifolium*, isolation and identification of five other known compounds from the leaves and stems, GC-MS analysis of the stem extract, and in silico studies with vicenins 1–3 (**3–5**) and related compounds for cancer chemopreventive and cancer chemotherapeutic potential.

## 2. Results and Discussion

**Figure 3:**
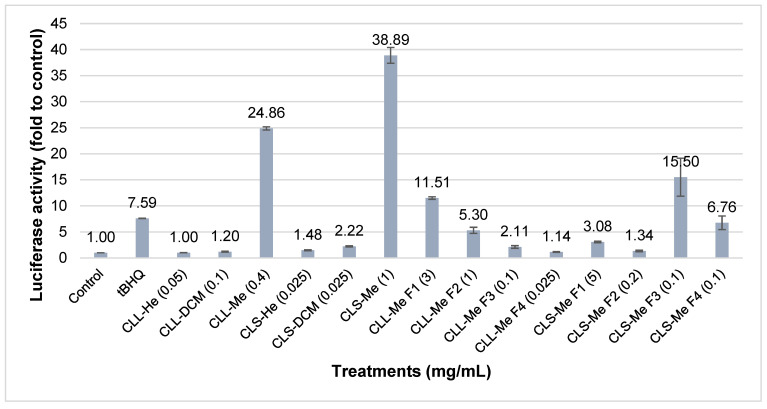
Induction of luciferase activity in AREc32 cells by crude extracts and fractions of *C. longifolium t*BHQ at 10 µM used as the positive control. The value of luciferase activity of untreated cells (control) was set at 1. Each bar represents the mean ± SEM of three independent experiments (5 replicates/experiment). CLL: *C. longifolium* leaves; CLS: *C. longifolium* stems, He: *n*-hexane; DCM: dichloromethane; Me: MeOH; F1–F4: fractions 1–4.

The *n*-hexane, dichloromethane (DCM), and methanol (MeOH) extracts obtained from leaves and stems of *C. longifolium* were evaluated for the ability to activate the Nrf2-ARE pathway using the luciferase reporter assay. Before conducting the luciferase reporter assay, the extracts with various concentrations (0.0125 to 1 mg/mL) were screened for their cytotoxicity in AREc32 cells by the MTT assay to determine a non-cytotoxic concentration of each extract (concentrations causing no more than 10% cell death). *t*BHQ at 10 μM, used as a positive control, caused a 6-fold activation of Nrf2 activity compared to the control. According to McMahon et al. [26], chemicals causing a >2-fold increase in the luciferase activity were defined as active. The MeOH extract of stems (1 mg/mL) was the most active with a 38.9-fold induction of luciferase activity (relative to control) followed by the MeOH extract of leaves (0.4 mg/mL) with a 24.9-fold induction and the DCM extract of stems (0.025 mg/mL) with a 2.2-fold induction (Figure 3). Fractionation of the active MeOH extracts followed by evaluation of their chemopreventive potential revealed MeOH fractions F1, F2 and F3 of the leaves and MeOH fractions F1, F3 and F4 of stems as active fractions, having a luciferase induction of more than 2-fold (Figure 3). Overall, upon fractionation by solid-phase extraction (SPE), the chemopreventive activity of MeOH fractions of leaves and stems was lower than respective crude extracts. In this case, the plausible explanation could be that the activities of the crude extracts resulted from the combined action of various compounds with synergistic or additive effects [27,28]. Moreover, in some cases, multiple constituents are required to observe the biological effect or get a better effect [29].

**Figure 4:**
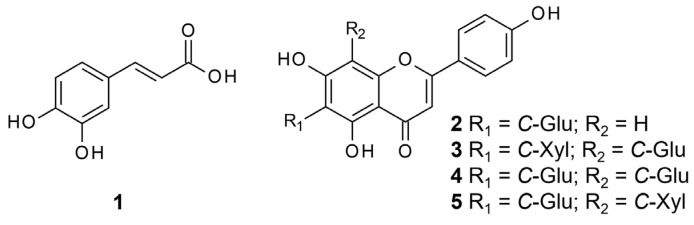
Structure of isolated compounds of *C. longifolium* leaves; Glu = glucosyl; Xyl = Xylosyl.

As the MeOH SPE fractions F1 and F2 of leaves and F3 and F4 of stems were overall the most active fractions, the phytochemical investigation was conducted on those active MeOH fractions of *C. longifolium* using a combination of reversed-phase solid-phase extraction (SPE) and preparative/semi-preparative/analytical HPLC techniques. The chromatographic separation of the active fractions F1 and F2 obtained from leaves led to the isolation and purification of five known compounds: a phenolic acid, caffeic acid (**1**) [30] and four *C*-glycosyl flavones, isovitexin (**2**) [31,32] and vicenins 1–3 (**3–5**) [33,34,35,36] (Figure 4), while the active fraction F4 of stems afforded hexadecanoic acid methyl ester (**6**). The structures of all isolated compounds were elucidated by extensive 1D and 2D NMR spectroscopic (^1^H, DEPTQ, COSY, NOESY, HSQC and HMBC) and mass spectrometric analyses, as well as by comparison with respective published spectral data in the literature.

**Figure 5:**
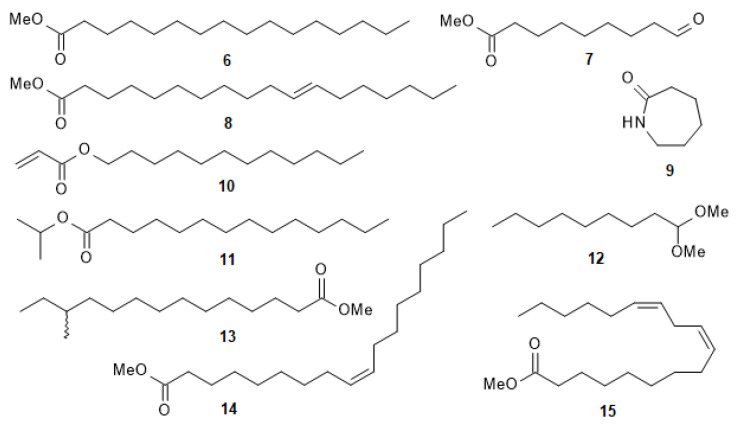
Structure of identified compounds of *C. longifolium* stems.

The chromatographic separation of the active MeOH fraction 4 of *C. longifolium* stems afforded a single compound, which was characterised as hexadecanoic acid methyl ester (**6**) by spectroscopic means (NMR and LC-MS) and by comparison of its spectral data with the published data [37]. GC-MS analysis and comparison with the National Institute of Standards and Technology (NIST) mass spectral library led to the tentative identification of ten phytochemicals in the active MeOH fractions F3 and F4 of *C. longifolium* stems including 9-oxononanoic acid methyl ester (**7**), 11-octadecenoic acid methyl ester (**8**), caprolactam (**9**), dodecyl acrylate (**10**), hexadecanoic acid methyl ester (**6**), isopropyl myristate (**11**), nonanal dimethyl acetal (**12**), 12-methyltetradecanoic acid methyl ester (**13**), (*Z*)-9-octadecenoic acid methyl ester (**14**) and (*Z,Z*)-9,12-octadecadienoic acid methyl ester (**15**) (Figure 5). Although caprolactam (**9**) was identified in this fraction, it is not a naturally occurring compound and is most likely a contaminant from leaching from plastic bags used for storing plant materials.

The tentatively identified compounds (by GC-MS) with their retention times, molecular formulas, molecular weights, and peak areas (%) are listed in Table 1. Thus, active MeOH fractions F3 and F4 of *C. longifolium* stems contained fatty acid methyl esters. Some of those compounds identified from the stems of *C. longifolium* have been reported to possess various biological activities. For instance, 11-octadecenoic acid methyl ester (**8**) and (*Z*)-9-octadecenoic acid methyl ester (**14**) were fatty acid methyl esters and have been revealed to exhibit cancer preventive activity [38,39]. 9-Oxononanoic acid methyl ester (**7**), (*Z,Z*)-9,12-octadecadienoic acid methyl ester (**15**), dodecyl acrylate (**10**) and hexadecanoic acid methyl ester (**6**) were previously acknowledged for anticancer [39,40,41,42,43,44] and antioxidant activities [44,45,46,47]. These compounds might contribute cancer chemopreventive effect of active MeOH fractions F3 and F4 of *C. longifolium* stems. Although fatty acid methyl esters occur in plants, the possibility of them being artefacts, especially when MeOH is used for extraction, cannot be ruled out. The identified compounds and biological activities related to medicinal uses are given in Table 2.

To the best of our knowledge, isolated compounds **1–6** and the GC-MS-assisted identified compounds **7–15** are reported here for the first time from *C. longifolium*. However, the nine phenolic compounds previously isolated from *C. longifolium* stems [26] were not found in the present study. It could be because the plant material was collected from different environmental conditions, affecting the profile of secondary metabolites [58]. Moreover, compound isolation was conducted from the most active fractions, not all fractions. This species belongs to the Euphorbiaceae family, which produces a wide range of secondary metabolites such as alkaloids, fatty acids, phenolic compounds, steroids and terpenoids [59,60,61].

**Figure 6:**
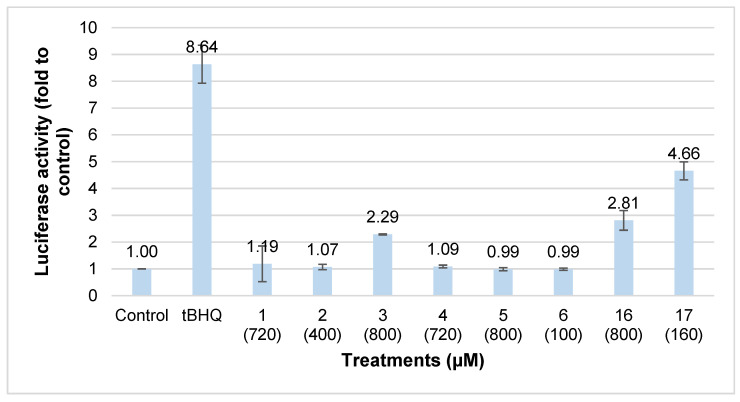
Induction of luciferase activity in AREc32 cells by compounds **1–6** and **16–17** *t*BHQ at 10 µM used as the positive control. The value of luciferase activity of untreated cells (control) was set at 1. Each bar represents mean ± SEM of three independent experiments (5 replicates/experiment).

The ability of compounds **1–6** to induce Nrf2 activation, correlating to their potential chemopreventive property, was determined by the luciferase reporter assay. AREc32 cells were incubated with each isolated compound at non-cytotoxic concentrations for 24 h. The luciferase activity of the isolated compounds is presented in Figure 6.

**Figure 7:**
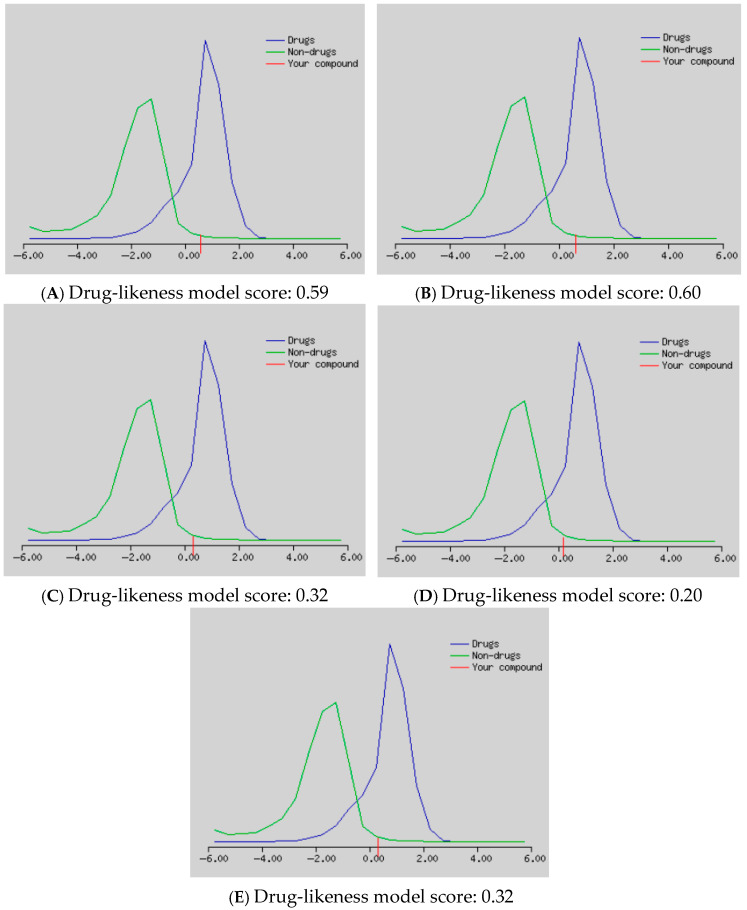
MolSoft drug-likeness score plots of (**A**) isovitexin (**2**), (**B**) vitexin (**16**), (**C**) vicenin 1 (**3**), (**D**) vicenin 2 (**4**), and (**E**) vicenin 3 (**5**); the blue-colored curve indicates a drug-like molecule and the green-colored curve indicates a non-drug-like molecule. The red-colored line indicates the score of the selected compounds.

Among the tested compounds, only vicenin 1 (**3**) at a non-cytotoxic concentration was considered a chemopreventive compound as it increased luciferase activity by 2.3-fold. To compare structure-function relationships, vitexin (**16**) and rosmarinic acid (**17**) were evaluated for their chemopreventive activities against AREc32 cells using the luciferase assay. The luciferase activity produced by the commercially obtained compounds **16** at 800 µM and **17** at 160 µM was 2.8 and 4.7-fold relative to the control, respectively (Figure 7). The similar features between compounds **16** and **3**, active flavone *C*-glycosides were the glucose unit attached to C-8 on the aglycone. This structural feature might be responsible for the activity. The rest of the isolated phenolic compounds showed no chemopreventive activity, which could be due to the glucose attachment to C-6 for compounds **2** and **5** and an additional glucose moiety at C-6 of compound **4**. Hence, the sugar position and the number of sugar moieties might affect the activity. Vicenin 1 (**3**), vitexin (**16**) and rosmarinic acid (**17**) have been proven to possess anti-inflammatory and antioxidant effects [62,63,64,65], which are intricately linked with cancer chemoprevention and chemotherapy [66].

In silico studies for bioactivity of five flavone *C*-glycosides, including isovitexin (**2**), vitexin (**16**), and vicenins 1–3 (**3–5**), were conducted, aiming to establish their drug-likeness properties and to predict the possible mechanism of action behind the cancer chemopreventive and chemotherapeutic effects. The PASS (prediction of activity spectra of substances) prediction analysis revealed the cancer chemopreventive and chemotherapeutic potentials of the selected *C*-glycosyl flavones (Table 3) as their Pa values exceeded 0.7. It was found that all the tested compounds showed high potential antineoplastic activity. Moreover, all the tested compounds except compound **16** were predicted to have enormous potential as anticarcinogenic. However, only compounds **24** and **16** had the potential as antimutagenic and apoptosis agonists. Additionally, only compounds **4** and **5** showed potential chemopreventive activity.

Drug-likeness analysis results of the tested compounds predicted by MolSoft are illustrated in Figure 7. Regarding drug-likeness scores, the compounds with positive values (nearer to 1) represent drug-like molecules [67]. Thus, all compounds showed good drug-likeness scores between 0.20 and 0.60. Vitexin (**16**) and isovitexin (**2**) had the best drug-likeness scores of 0.60 and 0.59, respectively.

The results of ADMET prediction for the tested compounds are presented in Table 4. Their bioavailability radar included six important physicochemical properties for oral bioavailability (lipophilicity, size, polarity, solubility, flexibility and saturation). In terms of size, vicenins 1–3 (**3–5**) exceeded the optimal size of the molecule (150–500 g/mol). All tested compounds exhibited a high degree of saturation, having fraction Csp3 values ranging from 0.29 to 0.44, which passed the filter of fraction Csp3 > 0.25. Furthermore, all tested compounds showed high polarity values, displaying topological polar surface area (TPSA) more than 130 Å^2^. Hence, these compounds were too polar and consequently predicted not to be orally bioavailable. Drugs with higher TPSA values are less lipid soluble [68]. For lipophilicity, the logP values of all compounds using a logP of a reference compound (XLOGP3) were in the optimal range (−0.7 to +5.0). The water solubility parameter is important for absorption. When three different topological models (ESOL, Ali and Silicos-IT) were applied, all compounds were predicted to be soluble to very soluble in water, meaning that they can be excreted in the kidneys and urine.

**Figure 8:**
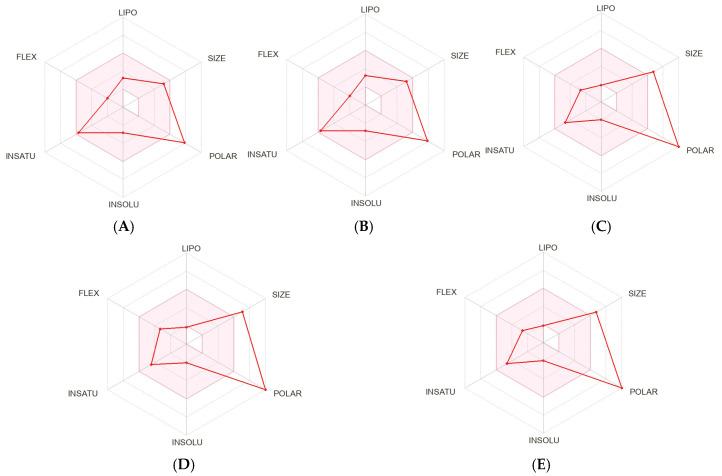
Bioavailability radar plots of (**A**) isovitexin (**2**), (**B**) vitexin (**16**), (**C**) vicenin 1 (**3**), (**D**) vicenin 2 (**4**), and (**E**) vicenin 3 (**5**); the pink area represents the optimal range for lipophilicity (LIPO: XLOGP3 −0.7 to +5.0, size (SIZE: MW 150–500 g/mol), polarity (POLAR: TPSA 20–130 Å^2^), solubility (INSOLU: log S < 6), saturation (INSATU: fraction Csp3 > 0.25) and flexibility (FLEX: rotatable bonds < 9); red line represents physicochemical properties of the compound.

In terms of pharmacokinetics, all compounds showed low gastrointestinal (GI) absorption. As a result, bioavailability is predicted to be poor. Notably, low oral bioavailability due to limited absorption is one of the issues found in flavonoids [69]. Alternatively, other administration routes, such as inhalation, injection, or the preparation of special dosage forms, such as nanoformulation, may require enhancing the bioavailability. Moreover, all compounds demonstrated no predictable blood-brain barrier (BBB) permeability, which means they were nontoxic or had a low toxicity to the brain. Additionally, none of the compounds were predicted to show any inhibition of CYP450 subtypes. This indicated that these compounds were not causing drug-drug interactions. Furthermore, negative log Kp values of the tested compounds revealed that all compounds exhibited low skin permeability. The medical chemistry parameters revealed that the tested compounds exhibited no structural alerts against PAINS (Pan-assay interference compounds) and Brenk’s filters. The summary of the prediction of oral bioavailability is plotted as the bioavailability radar with six parameters (Figure 8). Ideally, drug-likeness by this model requires the red lines assumed by the tested molecules to be entirely located in the pink zone of the plot [70]. Therefore, isovitexin (**2**), vitexin (**16**) and vicenins 1–3 (**3–5**) were predicted not to be orally bioavailable, because they were too polar and compounds **3–5** had high molecular weights.

**Figure 9:**
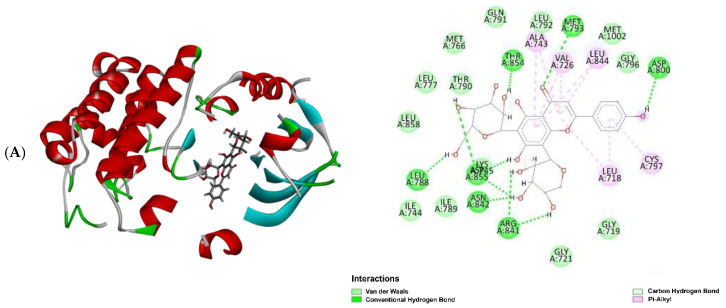

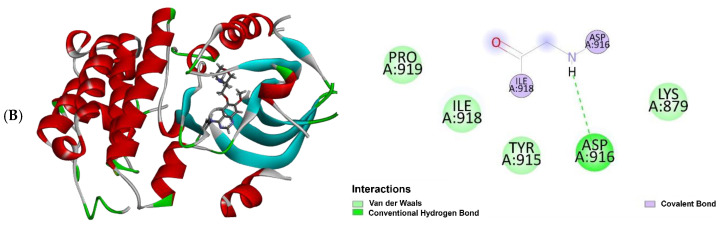
Molecular docking analysis: docking poses with 2D interactions of the best-docked compound and the positive control in the active pocket site of EGFR; (**A**) vicenin 3 (**5**) and (**B**) gefitinib.

**Figure 10:**
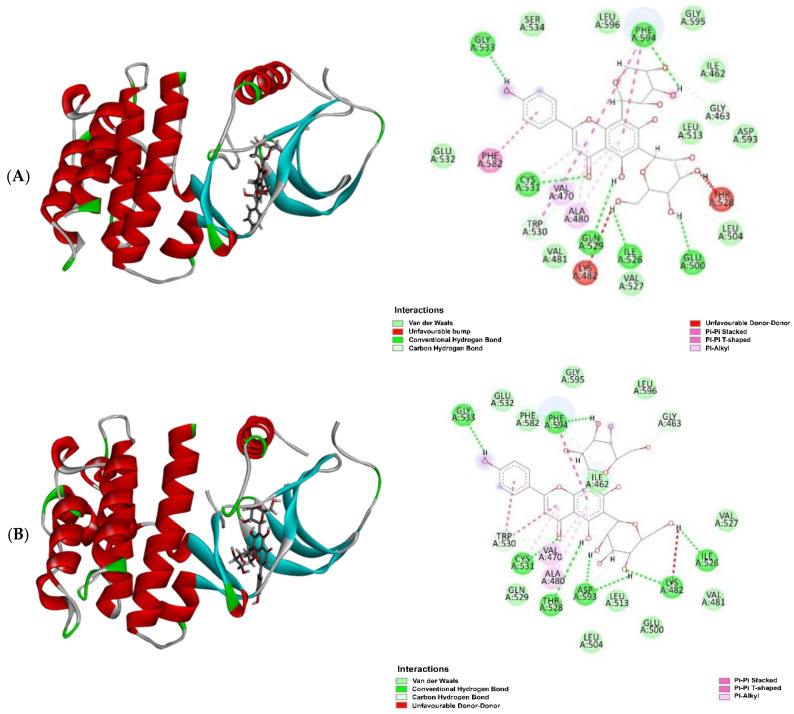

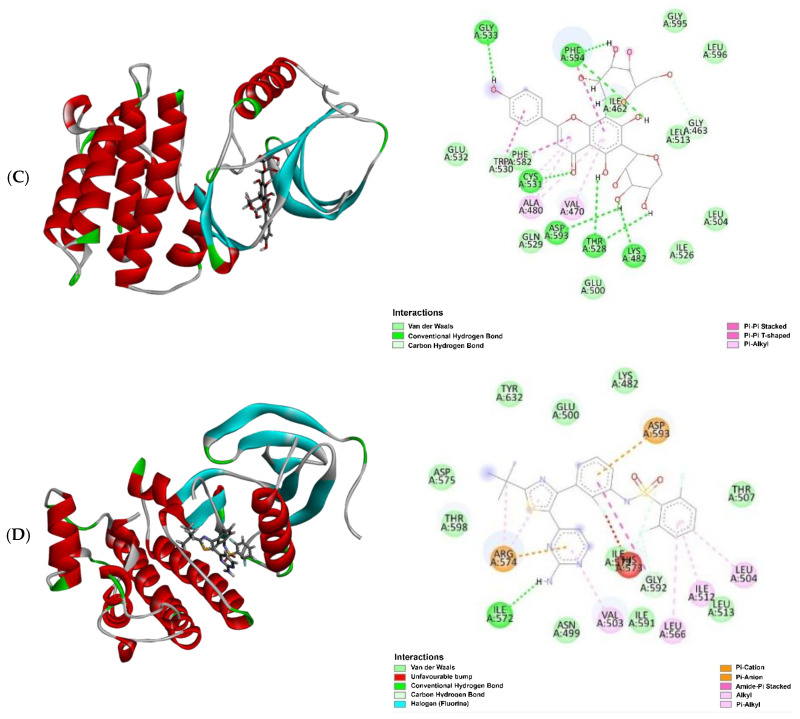
Molecular docking analysis: docking poses with 2D interactions of the best-docked compounds and the positive control in the active pocket site of BRAF; (**A**) vicenin 3 (**5**), (**B**) vicenin 2 (**4**), (**C**) vicenin 1 (**3**) and (**D**) dabrafenib.

**Figure 11:**
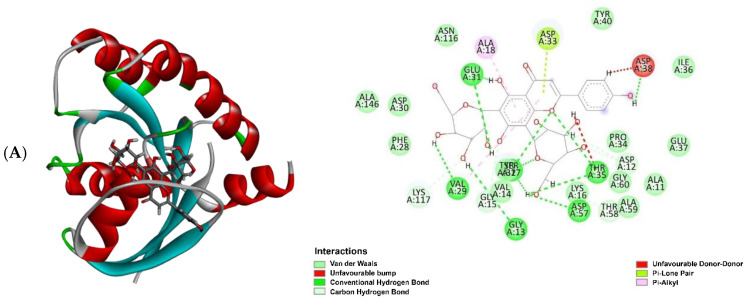

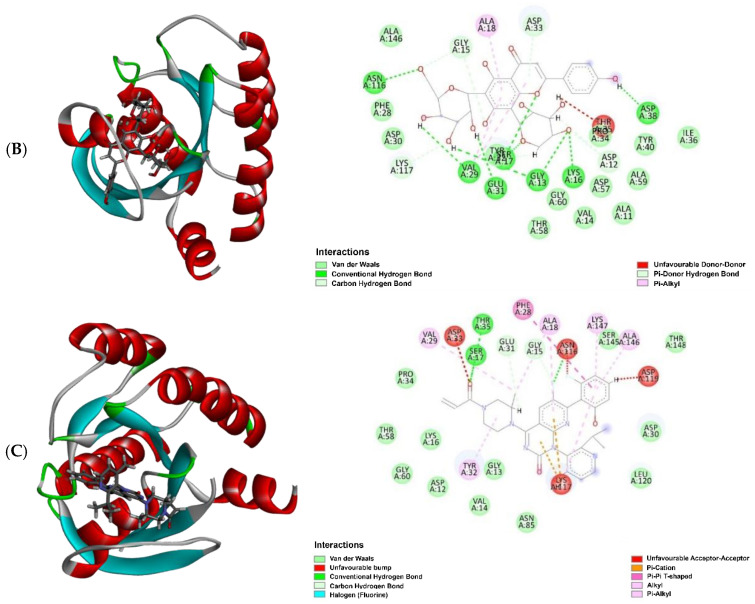
Molecular docking analysis: docking poses with 2D interactions of the best-docked compounds and the positive control in the active pocket site of KRAS; (**A**) vicenin 2 (**4**), (**B**) vicenin 3 (**5**) and (**C**) sotorasib.

**Figure 12:**
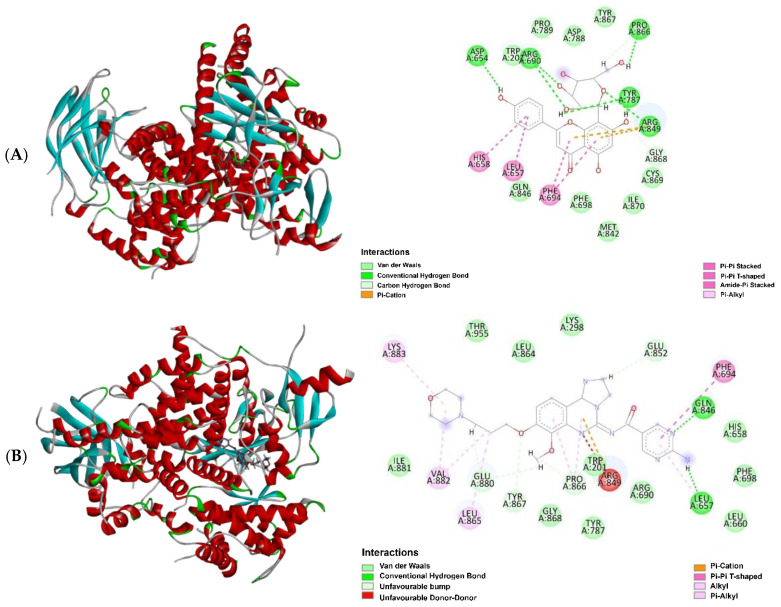
Molecular docking analysis: docking poses with 2D interactions of the best-docked compound and the positive control in the active pocket site of PI3K; (**A**) vitexin (**16**) and (**B**) copanlisib.

Molecular docking results revealed that the experimental compounds displayed significant contacts with the critical residues, demonstrating the ability of these proteins to alter their activity. Vicenin 3 (**5**) had the highest binding efficiency against three target proteins, except for PI3K, among all other compounds, including the positive controls (marketed drug). Docking analysis suggested the experimental compounds had cancer chemopreventive and chemotherapeutic potentials, whether it is involved with Nrf2 activation or other molecular mechanisms. If the top one compound is considered against each protein, compound **5** showed the best inhibition against EGFR and BRAF targets with a MolDock score of −142.57 and −162.63, respectively, whereas the positive control (marketed drug) gefitinib displayed a MolDock score of −141.80 and dabrafenib showed −137.99. Similarly, against KRAS and PI3K, the best inhibition was shown by vicenin 2 (**4**) and vitexin (**16**) with a MolDock score of −154.39 and −133.27, respectively, while the positive control sotorasib exhibited −150.41 and copanlisib showed −126.30. The entire MolDock scores of the experimental compounds against four cancer-related target proteins are displayed in Table 5. Docking pose and 2D interactions of best ligands and positive controls with target receptors are shown in Figure 9, Figure 10, Figure 11 and Figure 12.

**Figure 13:**
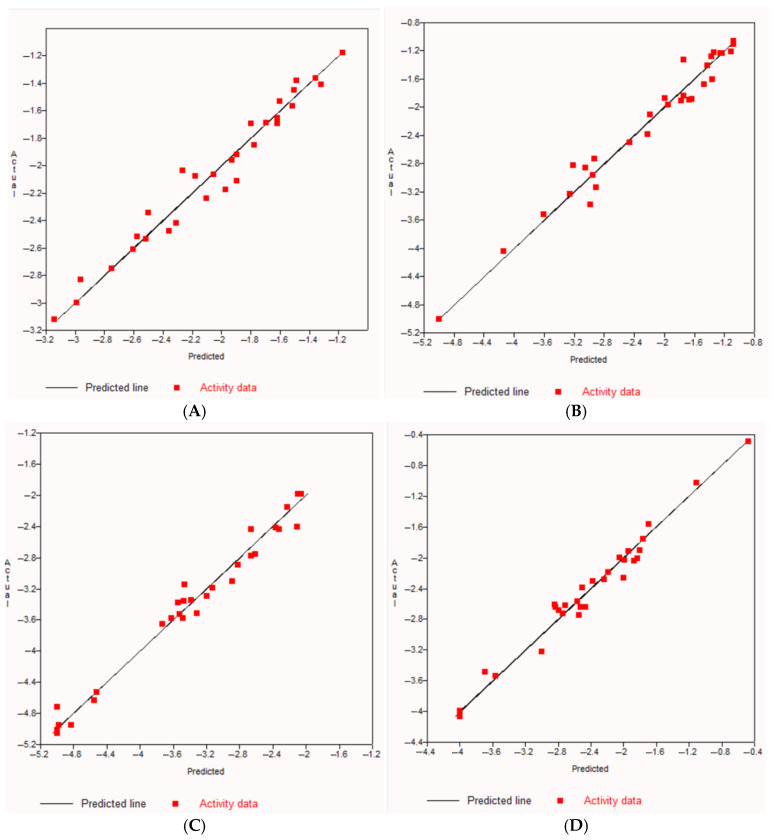
The multiple regression plots (linear) for the cancer-related target proteins: (**A**) EGFR, (**B**) BRAF, (**C**) KRAS and (**D**) PI3K.

QSAR models were used to acquire a better understanding of the structure–activity relationships. The prediction of biological activities was achieved quantitatively as a 50% inhibitory concentration (IC_50_) of a tested compound required to express a biological response. From the QSAR equation, the IC_50_ value of the experimental compounds of each target was calculated (Table 6). The multiple regression plots (linear) for the cancer-related target proteins are shown in Figure 13. From QSAR studies, it was found that the lowest IC_50_ value was shown by vicenin 2 (**4**) for the target EGFR as 2.45 nM. For the target BRAF, the lowest IC_50_ value was predicted to be 11.75 nM from vicenins 1 (**3**) and 3 (**5**), while the IC_50_ value of compound **4** was 58.88 nM. This means changing the type of sugar moieties of glycosylated compounds can make a difference in their activities [71,72]. The predicted IC_50_ value of vitexin (**16**) was 165.96 µM, considering the lowest value for the target KRAS. Compounds **3** and **5** also showed the lowest IC_50_ value (64.57 nM) for the target PI3K. These results were a supporting indicator if we consider vicenins 1 (**3**) and 3 (**5**) as potential contributors to anticancer activity.

In silico studies and literature reviews suggested that these flavone *C*-glycosides have potential as therapeutically acceptable cancer chemotherapeutic agents. Moreover, these compounds also have cancer chemopreventive prospects. In silico study results supported the reported potential anticancer properties of these compounds. However, extensive in vitro or in vivo preclinical studies are required, as well as clinical trials. Although, the in silico studies showed the cancer chemopreventive activity profiles of isovitexin (**2**), vitexin (**16**) and vicenins 1–3 (**3–5**), when in vitro chemopreventive activity using the luciferase assay was performed, compounds **2**, **4** and **5** were not active against AREc 32 cells in terms of activation of Nrf2 activity. The in vitro luciferase assay results of compounds **3** and **16** found that these compounds induced Nrf2 activation in AREc32 cells. Therefore, the results of the in vitro studies matched the findings from the in silico work. It demonstrates the need for conducting in vitro and in vivo studies together with in silico studies. In silico is a prediction based on numerous factors such as molecular structure, physicochemical, and pharmacokinetic properties, but the reality may be different from in silico prediction. Thus, in silico prediction is not always 100% accurate. Even if the results from the in vitro cancer chemopreventive effect indicated that compounds **4** and **5** were not Nrf2 activators, it does not mean that compounds **4** and **5** do not exert a chemopreventive effect. These two compounds may be contributing to cancer chemoprevention through other signalling pathways, not necessarily through Nrf2 activation.

Apart from the Nrf2/ARE signalling pathway, there are many other cellular signalling pathways that play important roles in cancer chemoprevention [2]. In recent years, growing evidence has revealed the significance of natural products in affecting the numerous essential cellular signalling pathways associated with cancer such as activator protein-1 (AP-1), Janus kinase/signal transducers and activators of transcription (JAK/STAT), mitogen-activated protein kinase (MAPK), nuclear factor-Kappa B (NF-κB), phosphatidylinositol 3-kinase/protein kinase B/mammalian target of rapamycin (PI3K-AKT-mTOR) pathways [73]. Notably, these natural products prevent cancer cell proliferation, development, and metastasis by inducing cell cycle arrest, promoting apoptosis, generating reactive oxygen species (ROS), inducing/inhibiting several signalling pathways, inhibiting angiogenesis and regulating transcription factors [74].

## 3. Materials and Methods

### 3.1. Chemicals, Reagents, and Materials

All chemicals were purchased from Sigma-Aldrich (Dorset, UK). Solvents were purchased from Fisher Scientific (Loughborough, UK) and used without further purification. Deuterated NMR solvents, including methanol (CD_3_OD), pyridine (Pyr-d_5_) and DMSO (DMSO-d_6_), were purchased from Cambridge Isotope Laboratories (Tewksbury, MA, USA). Cell culture reagents (0.25% trypsin-EDTA, penicillin-streptomycin and phosphate-buffered saline (PBS)) were purchased from Thermo Fisher Scientific (Paisley, UK), while basal medium (DMEM), fetal bovine serum (FBS) and geneticin G418 were purchased from Biosera (Neuville, France). Luciferase assay kit reagent was purchased from Promega (Madison, WI, USA). Specialized white 96-well flat-bottom plates were from Greiner Bio-One (Frickenhausen, Germany), while clear 96-well flat-bottom plates were from Falcon (Falcon, NC, USA).

### 3.2. General Experimental Procedures

Analytical HPLC was performed on a Dionex Ultimate 3000 UHPLC (Thermo Fisher Scientific, Waltham, MA, USA) coupled with a diode array detector (DAD). MeOH crude extracts and MeOH fractions were analyzed on a reversed-phase Phenomenex column (Luna 5 μm C18(2) 100 Å, 150 mm × 4.6 mm, serial no. H21-192754, Phenomenex, Torrance, CA, USA). Preparative HPLC system of a Dionex Ultimate 3000 UHPLC (Thermo Fisher Scientific, MA, USA) or a Jasco LC-4000 Series (Jasco, Tokyo, Japan) and a reverse phase Phenomenex column (Luna 10 μm C18(2) 100 Å, 150 mm × 21.2 mm, serial no. H18-196260, Phenomenex, CA, USA) were used to isolate compounds from active fractions. The purification of impure compounds was accomplished on an Agilent 1260 Infinity series semi-preparative HPLC system coupled with a DAD detector using an ACE 10 C18-HL column (150 mm × 10 mm, Hichrom Ltd., Lutterworth, UK). A flow rate of 1 mL/min, 2 mL/min and 10 mL/min was used for analytical, semi-preparative and preparative HPLC, respectively. The column temperature was maintained at 25 °C. The chromatogram was monitored at variable UV-vis wavelengths (220, 254, 280 and 320 nm). The NMR experiments were performed in CD_3_OD, Pyr-d_5_ or DMSO-d_6_ solution on a Bruker AMX-600 NMR spectrometer (600 MHz for ^1^H, and 150 MHz for ^13^C) (Bruker, Billerica, MA, USA). ESI-MS data were recorded through a Micromass LCT time-of-flight (TOF) mass spectrometer (Waters, Manchester, UK) equipped with an electrospray ionisation (ESI) source. The GC-MS analysis of oily fractions of *C. longifolium* stems was performed using an Agilent 6890N network GC system equipped with an Agilent 5790 mass selective detector (Agilent, Santa Clara, CA, USA) and a capillary column HP-5MS (30 m × 0.25 mm i.d., 0.25 μm film thickness). The oven temperature was programmed as follows: the beginning of 150 °C kept for 3 min, rising by 20 °C/min to 280 °C and maintaining for 4 min. The run time was 14 min. The mass-spectrometric analysis was conducted in the range of 41–500 *m*/*z* using electronic ionisation energy, the EI mode at 70 eV. The MS source temperature was 230 °C. Identification of the detected components was performed by comparing their mass spectra with the reference spectra in NIST MS Search software version 3.0.

### 3.3. Plant Materials

Leaves (CLL) and stems (CLS) of *Claoxylon longifolium* (Blume) Endl. ex Hassk. were collected by Dr Chuanchom Khuniad from Phatthalung Province, southern Thailand (Latitude: 7°37′4.30″ N; Longitude: 100°04′40.51″ E), during April and May 2021. Its voucher specimen (BKF NO.228283) was prepared and deposited in the Forest Herbarium (BKF), Department of National Park, Wildlife and Plant Conservation, Ministry of Natural Resources, Bangkok, Thailand.

### 3.4. Extraction and Isolation of Compounds

Leaves (278.0 g) and stems (473.0 g) of *C. longifolium* were washed, sliced into small pieces, and dried in a solar greenhouse dryer at a temperature not exceeding 55 °C. Dried leaves and stems were ground into powder with a spice grinder. Powdered leaves and stems were Soxhlet-extracted separately and consecutively with *n*-hexane, dichloromethane (DCM) and methanol (MeOH) (900 mL, ten cycles each) [75]. The resulting extracts were concentrated separately to dryness under reduced pressure using a rotary evaporator at a maximum temperature of 45 °C (BUCHI, Flawil, Switzerland). The crude extracts at a non-cytotoxic concentration were screened for their chemopreventive potential using a luciferase reporter assay in AREc32 cells. MeOH extracts of leaves and stems were found to be the most active extracts. A portion of each active MeOH extract (2 g) was subjected to solid-phase extraction (SPE) on a Strata C18-E reversed-phase cartridge (20 g) (Phenomenex, CA, USA), previously washed with MeOH (50 mL), followed by equilibration with water (100 mL) [76]. The SPE cartridge was eluted with a step gradient consisting of 20, 50, 80% MeOH in water and 100% MeOH (200 mL each) to obtain four MeOH fractions (F1–F4), respectively. All fractions were concentrated to dryness by a rotary evaporator combined with a nitrogen blowdown concentrator and kept at 4 °C for further analyses. MeOH fractions F1 and F2 of leaves (CLL-Me F1 and F2) were analyzed by preparative HPLC (Jasco) with a mobile phase gradient of water (A) and methanol (B), both containing 0.1% TFA: 30–50% A in B for 40 min, monitored at wavelength 220 nm. Preparative HPLC analysis of CLL-Me F1 using the above method yielded caffeic acid (**1**, 5.5 mg, *t*_R_ = 24.7 min). The separation process of CLL-Me F2 afforded vicenin 2 (**4**, 13.1 mg, *t*_R_ = 22.8 min) and isovitexin (**2**, 25.3 mg, *t*_R_ = 34.1 min). Furthermore, CLL-Me F2-P3 (15.8 mg) collected at *t*_R_ = 26.3 min was further purified by semi-preparative HPLC with a mobile phase gradient of 33–45% A in B over 20 min, observed at wavelength 220 nm to afford vicenin 3 (**5**, 7.5 mg, *t*_R_ = 15.2 min). CLL-Me F2-P5 (18.7 mg) collected at *t*_R_ =32.1 min was also purified by semi-preparative HPLC with a different mobile phase: a gradient of 40–55% A in B over 20 min, observed at wavelength 220 nm to afford vicenin 1 (**3**, 9.7 mg, *t*_R_ = 13.8 min).

The MeOH fraction F3 of stems (CLS-Me F3) was separated by preparative HPLC (Dionex) with a mobile phase gradient of 45–100% A in B for 35 min, observed at wavelength 220 nm to yield CLS-Me F3-P3 (14.4 mg, *t*_R_ = 30.4 min). Fraction F4 of stems (CLS-Me F4) was also separated by preparative HPLC (Dionex) with mobile phase gradient of 75–100% A in B for 35 min, observed at wavelength 220 nm to obtain CLS-Me F4-P6 (25.4 mg, *t*_R_ = 26.7 min), CLS-Me F4-P7 (34.4 mg, *t*_R_ = 29.6 min), CLS-Me F4-P8 (38.7 mg, *t*_R_ = 32.1 min) and CLS-Me F4-P9 (14.6 mg, *t*_R_ = 33.3 min). CLS-Me F4-P8 was further purified by analytical HPLC with a mobile phase gradient of 85–100% A in B for 20 min. This purification method obtained hexadecanoic acid methyl ester (**6**, 14.3 mg, *t*_R_ = 14.7 min). CLS-Me F3-P3, CLS-Me F4-P6, CLS-Me F4-P7 and CLS-Me F4-P9 (oily fractions) were found to be a mixture of fatty acids, long-chain alkanes and others by analytical HPLC and preliminary ^1^H NMR. Hence, they were not further purified. However, the GC-MS analysis was conducted to identify the presence of phytochemicals in CLS-Me F3-P3, CLS-Me F4-P6, CLS-Me F4-P7, compound **6** and CLS-Me F4-P9.

### 3.5. Cell Culture

An AREc32 cell line (a modified MCF7 human breast cancer cell line) was obtained from Professor Roland Wolf (University of Dundee, UK), whose lab created the cell line. AREc32 cells were cultured in Dulbecco’s Modified Eagle’s Medium (DMEM) High Glucose supplemented with 10% fetal bovine serum (FBS), 1% penicillin-streptomycin and 0.8 mg/mL of antibiotic G418 in T75 cell culture flasks and maintained at 37 °C in a humidified atmosphere of 5% CO_2_ (Binder, Tuttlingen, Germany).

### 3.6. MTT Assay

The cell viability of the AREc32 cells was assessed using the MTT assay [77] to identify a non-cytotoxic concentration for further use in the luciferase reporter assay. The cells were seeded into 96-well plates at a density of 1.2 × 10^4^ cells/well (100 μL/well) and allowed to adhere for 24 h in the incubator at 37 °C and 5% CO_2_. After 24 h, the medium was removed, and the cells were treated with medium containing different concentrations of each treatment (crude extracts, fractions and compounds). A set of untreated control wells was included in each plate, as well as cells treated with 20% DMSO as a positive control. After incubation with the treatment for 24 h, the medium was aspirated. A volume of 20 μL of MTT solution (5 mg/mL in PBS) and 180 μL of fresh medium were added to each well and then continued to incubate for 4 h. The MTT solution/medium was aspirated off and replaced with DMSO (100 μL/well) to dissolve the purple formazan crystals. The absorbance of the formazan crystals was then read using a microplate reader at 570 nm (TECAN, Mannedorf, Switzerland). All experiments were performed in three independent experiments (5 replicates/experiment). The mean % cell viability was calculated as follows:

$$Cell\;viability=(A_{1}/A_{0})\times 100$$

where A_0_ is the mean absorbance of untreated wells and A_1_ is the mean absorbance of treated cells.

### 3.7. Luciferase Reporter Assay

To investigate the cancer chemopreventive potential, a luciferase reporter assay with an AREc32 cell line was used to assess the ability of the crude extracts, fractions, and compounds to induce the cellular antioxidant defence system via the activation of Nrf2 transcription factor [78]. Briefly, AREc32 cells were seeded into 96-well plates at a density of 1.2 × 10^4^ cells/well and incubated for 24 h. They were then treated with a non-cytotoxic concentration of each crude extract, fraction and compound. After another 24 h of incubation, the culture medium was discarded, and the cells were washed with 100 μL of phosphate-buffered saline (PBS) twice, then 20 μL of 1X luciferase reporter lysis buffer was added to each well, followed by a freeze-thaw cycle (−20 °C) for 24 h to achieve complete cell lysis. The cell lysate was thawed and transferred into a white opaque 96-well plate and mixed with 100 μL of freshly prepared luciferase assay reagent and immediately. The luminescence was measured immediately using the microplate reader (TECAN, Mannedorf, Switzerland). The luciferase activity level was compared to the basal level of luciferase activity in control cells and presented as a fold increase relative to control (untreated cells). *Tert*-butylhydroquinone (*t*BHQ, 10 μM) served as a positive control. All experiments were performed in three independent experiments (5 replicates/experiment).

### 3.8. In Silico Studies for Bioactivity of Selected C-Glycosyl Flavones

Five *C*-glycosyl flavones, including vitexin (**16**), isovitexin (**2**), and vicenins 1–3 (**3–5**), were selected for in silico studies using several screening methods and protocols as follows:

Prediction of activity spectra for substances (PASS) analysis of biological activities:

The PASS online (http://www.way2drug.com/passonline/predict.php, accessed on 20 August 2024) was utilized to predict the probability of the selected compounds exhibiting six biological activities related to cancer or being inactive [79].

Drug likeness analysis:

Drug likeness properties of the *C*-glycosyl flavones were screened by the online MolSoft toolkit (https://molsoft.com/mprop/, accessed on 20 August 2024).

Adsorption, distribution, metabolism, excretion and toxicity (ADMET) prediction:

Prediction of ADMET profiles was analyzed by the SwissADME server (http://www.swissadme.ch/), a web server from Swiss Institute of Bioinformatics [70], to evaluate the physicochemical, pharmacokinetics, drug-likeness, and medicinal chemistry friendliness parameters of the selected *C*-glycosyl flavones.

Molecular docking:

Molecular docking was analyzed with the Molegro Virtual Docker 6.0 (MVD) software version 6.0 [80,81] for predicting the binding interactions of ligands (selected compounds) with the active sites of cancer-related target proteins (EGFR, BRAF, KRAS, and PI3K). EGFR and BRAF are associated with uncontrolled cell proliferation, while KRAS and PI3K are responsible for anti-apoptotic activity.

Quantitative structure–activity relation (QSAR) studies:

QSAR studies were conducted to find relationships between the structural properties of the selected compounds and cancer-associated biological activities by considering 30 known inhibitors of each target protein from the ChEMBL database (https://www.ebi.ac.uk/chembl/). ACD ChemSketch software (Advanced Chemistry Development, Toronto, ON, Canada) was used to create 11 descriptors from each inhibitor, including molar weight, molar refractivity, molar volume, index of refraction, surface tension, parachor, density, polarizability, log P, hydrogen bond donor and hydrogen bond acceptor. The Easy QSAR software developed by Kuntal Kumar Bhusan, University of Hyderabad, was used to create a multiple linear regression analysis after the descriptors and activities were entered. This regression study produced the QSAR equation. The 50% inhibitory concentration (IC_50_) values of the *C*-glycosyl flavones of each target were calculated by the QSAR equation.

### 3.9. Statistical Analysis

All experiments were conducted in triplicate with a minimum of three replicates per assayed concentration. Data were expressed as means ± SEM (standard error of the mean). The graphs were plotted using Microsoft Excel for Microsoft 365 MSO Version 2502. SEM was shown on the graph as an error bar.

## 4. Conclusions

*Claoxylon longifolium* (leaves and stems) has been evaluated for its phytochemicals and cancer chemopreventive effect following a bioassay-guided isolation procedure. The phytochemical investigation conducted on *C. longifolium* active MeOH fractions afforded six known compounds: caffeic acid (**1**), isovitexin (**2**), and vicenins 1–3 (**3–5**), as well as hexadecanoic acid methyl ester (**6**). GC-MS analysis revealed that the major constituents present in the active stem fractions were fatty acid methyl esters. Vicenin 1 (**3**) was found to be a potent chemopreventive compound as it increased luciferase activity by 2.3-fold. In silico studies on the *C*-glycosyl flavones revealed the potential of these compounds as cancer chemopreventive and chemotherapeutic agents. This study generated the first phytochemical investigation on *C. longifolium* leaves. All identified and isolated compounds were reported for the first time from this species. Moreover, this is also the first report on the chemopreventive properties of *C. longifolium*. The findings of a recent study indicate that *C. longifolium* contains promising chemopreventive agents, supporting, to some extent, the utilization of this indigenous vegetable in cancer prevention and consumption for health benefits in the southern Thai community.

## Figures and Tables

**Table 1:** Phytochemicals identified in collected peaks and a purified compound obtained from active MeOH fractions 3 and 4 of *C. longifolium* stems by GC-MS.

**Sample**	**Retention Time (*t*_R_) in min**	**Molecular Formula**	**Molecular Weight**	**Compound Name**	**Peak Area** **(%)**
CLS-Me F3-P3	6.03	C_15_H_28_O_2_	240.21	Dodecyl acrylate (**10**)	50.47
CLS-Me F4-P6	2.60	C_6_H_11_NO	113.08	Caprolactam (**9**)	51.97
	3.97	C_10_H_18_O_3_	186.13	9-Oxononanoic acid methyl ester (**7**)	13.69
	5.32	C_11_H_24_O_2_	188.18	Nonanal dimethyl acetal (**12**)	13.07
	6.26	C_16_H_32_O_2_	256.24	12-methyltetradecanoic acid methyl ester (**13**)	6.29
	6.91	C_17_H_34_O_2_	270.26	Isopropyl myristate (**11**)	4.72
CLS-Me F4-P7	3.98	C_10_H_18_O_3_	186.13	9-Oxononanoic acid methyl ester (**7**)	8.32
	6.93	C_17_H_34_O_2_	270.26	Isopropyl myristate (**11**)	21.93
	8.42	C_19_H_34_O_2_	294.26	(*Z,Z*)-9,12-octadecadienoic acid methyl ester (**15**)	33.84
Compound **6**	7.50	C_17_H_34_O_2_	270.26	Hexadecanoic acid methyl ester (**6**)	100.00
CLS-Me F4-P9	2.63	C_6_H_11_NO	113.08	Caprolactam (**9**)	13.68
	6.93	C_17_H_34_O_2_	270.26	Isopropyl myristate (**11**)	4.44
	7.51	C_17_H_34_O_2_	270.26	Hexadecanoic acid methyl ester (**6**)	10.15
	8.49	C_19_H_36_O_2_	296.27	(*Z*)-9-octadecenoic acid methyl ester (**14**)	61.56
	8.49 (overlapping peak on right)	C_19_H_36_O_2_	296.27	11-Octadecenoic acid methyl ester (**8**)	

**Table 2:** Biological activity of identified phytochemicals from active MeOH fractions 3 and 4 of *C. longifolium* stems.

Compound Name	Biological Activity	References
9-Oxononanoic acid methyl ester (**7**)	Anti-acne, antifungal, antihyperpigmentative, anti-inflammatory, antileukemic, antimicrobial, antioxidant, antiproliferative, antitumor, cytotoxic and larvicidal	[39,46]
11-Octadecenoic acid methyl ester (**8**)	5α-Reductase inhibitor, anti-acne, antiandrogenic, Antiarthritic, antibacterial, anticoronary, antidiarrheal, antieczemic, antihypercholesterolemia, anti-inflammatory, cancer preventive and hepatoprotective	[39,42]
Caprolactam (**9**)	Antibacterial	[48]
Dodecyl acrylate (**10**)	Antibacterial, anticancer, antifungal and antioxidant	[41,43,45,49]
Hexadecanoic acid methyl ester (**6**)	5α-Reductase inhibitor, anti-acne, anti-androgenic, antiarthritic, antibacterial, anticancer, anticoronary, antieczemic, antihistaminic, anti-inflammatory, antioxidant, hepatoprotective, hypocholesterolemic, insecticide and nematicide	[38,47]
Isopropyl myristate (**11**)	Antifungal and antimicrobial	[50,51]
Nonanal dimethyl acetal (**12**)	Antibacterial	[52]
12-Methyltetradecanoic acid methyl ester (**13**)	Antifungal	[53]
(*Z*)-9-Octadecenoic acid methyl ester (**14**)	5α-Reductase inhibitor, anemiagenic anti-androgenic, antidiarrheal, anti-inflammatory, antimicrobial, cancer preventive, dermatitigenic, hypocholesterolemic and insectifuge	[38,44,54,55,56,57]
(*Z,Z*)-9,12-Octadecadienoic acid methyl ester (**15**)	Anti-arthritic, anticancer, anti-eczemic, antifungal, antihistaminic, anti-inflammatory, antioxidant, antitumour, hepatoprotective and hypocholesterolemic	

**Table 3:** PASS prediction analysis of the selected C-glycosyl flavones.

Predicted Bioactivities	Isovitexin (2)		Vitexin (16)		Vicenin 1 (3)		Vicenin 2 (4)		Vicenin 3 (5)	
	Pa	Pi	Pa	Pi	Pa	Pi	Pa	Pi	Pa	Pi
Anticarcinogenic	0.856	0.004	-	-	0.872	0.003	0.831	0.004	0.872	0.003
Antimutagenic	0.795	0.004	0.820	0.004	-	-	-	-	-	-
Antineoplastic	0.800	0.012	0.836	0.008	0.852	0.007	0.833	0.008	0.852	0.007
Apoptosis agonist	0.706	0.014	0.737	0.012	-	-	-	-	-	-
Chemopreventive	-	-	-	-	-	-	0.859	0.003	0.905	0.002
Proliferative disease treatment	-	-	0.723	0.005	0.720	0.005	-	-	0.720	0.005

Pa = Probability to be active; Pi = Probability to be inactive.

**Table 4:** The SwissADME predicted physicochemical, pharmacokinetic, drug-likeness, and medicinal chemistry properties of the selected C-glycosyl flavones.

Compounds	2	3	4	5	16
**Physicochemical properties**					
Molecular weight (g/mol)	432.38	564.49	594.52	564.49	432.38
Heavy atoms	31	40	42	40	31
Aromatic heavy atoms	16	16	16	16	16
Fraction Csp3	0.29	0.42	0.44	0.42	0.29
Rotatable bonds	3	4	5	4	3
H-bond acceptors	10	14	15	14	10
H-bond donors	7	10	11	10	7
Molar refractivity (MR)	106.61	133.26	139.23	133.26	106.61
TPSA (Å^2^)	181.05	250.97	271.2	250.97	181.05
**Lipophilicity**					
Log Po/w (iLOGP)	1.94	1.41	1.27	1.63	1.38
Log Po/w (XLOGP3)	0.21	−2.19	−2.26	−2.19	0.21
Log Po/w (WLOGP)	−0.23	−2.4	−3.04	−2.4	−0.23
Log Po/w (MLOGP)	−2.02	−3.97	−4.51	−3.97	−2.02
Log Po/w (Silicos-IT)	0.33	0.33	−1.8	0.33	0.33
Consensus Log P	−0.07	−1.7	−2.07	−1.65	0.05
**Water solubility**					
Log S (ESOL)	−2.84	−1.99	−2.05	−1.99	−2.84
ESOL solubility (mg/mL)	6.29 × 10^−1^	5.75	5.25	5.75	6.29 × 10^−1^
ESOL solubility (mol/L)	1.46 × 10^−3^	1.02 × 10^−2^	8.83 × 10^−3^	1.02 × 10^−2^	1.46 × 10^−3^
ESOL class	Soluble	Very soluble	Soluble	Very soluble	Soluble
Log S (Ali)	−3.57	−2.55	−2.9	−2.55	−3.57
Ali solubility (mg/mL)	1.16 × 10^−1^	1.59	7.46 × 10^−1^	1.59	1.16 × 10^−1^
Ali solubility (mol/L)	2.68 × 10^−4^	2.82 × 10^−3^	1.26 × 10^−3^	2.82 × 10^−3^	2.68 × 10^−4^
Ali class	Soluble	Soluble	Soluble	Soluble	Soluble
Log S (Silicos-IT)	−2.38	−0.71	−0.27	−0.71	−2.38
Silicos-IT solubility (mg/mL)	1.81	1.10 × 10^2^	3.19 × 10^2^	1.10 × 10^2^	1.81
Silicos-IT solubility (mol/L)	4.20 × 10^−3^	1.95 × 10^−1^	5.36 × 10^−1^	1.95 × 10^−1^	4.20 × 10^−3^
Silicos-IT class	Soluble	Soluble	Soluble	Soluble	Soluble
**Pharmacokinetics**					
GI absorption	Low	Low	Low	Low	Low
BBB permeant	No	No	No	No	No
P-gp substrate	No	Yes	No	Yes	No
CYP1A2 inhibitor	No	No	No	No	No
CYP2C19 inhibitor	No	No	No	No	No
CYP2C9 inhibitor	No	No	No	No	No
CYP2D6 inhibitor	No	No	No	No	No
CYP3A4 inhibitor	No	No	No	No	No
Log Kp (cm/s)	−8.79	−11.3	−11.53	−11.3	−8.79
**Drug likeness**					
Lipinski violations	1	3	3	3	1
Ghose violations	0	3	4	3	0
Veber violations	1	1	1	1	1
Egan violations	1	1	1	1	1
Muegge violations	2	4	4	4	2
Bioavailability score	0.55	0.17	0.17	0.17	0.55
**Medicinal chemistry**					
PAINS alerts	0	0	0	0	0
Brenk alerts	0	0	0	0	0
Leadlikeness violations	1	1	1	1	1
Synthetic accessibility	4.99	6.18	6.4	6.18	5.12

**Table 5:** MolDock scores of the ligands for their cancer-related target proteins.

**Target Protein: EGFR**			**Target Protein: BRAF**		
**Ligand**	**MolDock Score**	**HBond**	**Ligand**	**MolDock Score**	**HBond**
Vicenin 3 (**5**)	−142.57	−14.34	Vicenin 3 (**5**)	−162.63	−14.88
Gefitinib *	−141.80	−3.22	Vicenin 2 (**4**)	−144.19	−12.17
Vicenin 1 (**3**)	−141.69	−15.00	Vicenin 1 (**3**)	−143.07	−7.57
Vicenin 2 (**4**)	−138.86	−9.31	Dabrafenib *	−137.99	−3.27
Vitexin (**16**)	−138.07	−12.85	Vitexin (**16**)	−134.15	−4.44
Isovitexin (**2**)	−109.20	−14.82	Isovitexin (**2**)	−132.29	−10.80
**cin: KRAS**			**Target protein: PI3K**		
**Ligand**	**MolDock score**	**HBond**	**Ligand**	**MolDock score**	**HBond**
Vicenin 2 (**4**)	−154.39	−18.82	Vitexin (**16**)	−133.27	−12.62
Vicenin 3 (**5**)	−153.37	−17.17	Copanlisib *	−126.30	−6.37
Sotorasib *	−150.41	−3.61	Isovitexin (**2**)	−85.47	−11.64
Vicenin 1 (**3**)	−149.67	−17.55	Vicenin 1 (**3**)	−75.69	−13.35
Vitexin (**16**)	−149.03	−11.27	Vicenin 2 (**4**)	−71.60	−14.93
Isovitexin (**2**)	−140.77	−12.91	Vicenin 3 (**5**)	−67.12	−15.75

* Positive control.

**Table 6:** The predicted IC_50_ (nm) values of the experimental compounds using the QSAR model for four cancer-related target proteins.

Compounds	Calculated IC_50_			
	EGFR (nM)	BRAF (nM)	KRAS (µM)	PI3K (nM)
**2**	15.85	28.18	169.82	245.47
**3**	7.08	11.75	186.21	64.57
**4**	2.45	58.88	229.09	363.08
**5**	7.08	11.75	186.21	64.57
**16**	18.62	60.26	165.96	213.80

## Data Availability

All data are available from the corresponding authors and may be shared upon a formal written request.
